# Functional dyspepsia: A new approach from traditional Persian medicine 

**Published:** 2016

**Authors:** Mehdi Pasalar, Majid Nimrouzi, Rasool Choopani, Mahmoud Mosaddegh, Mohammad Kamalinejad, Abdolali Mohagheghzadeh, Kamran Bagheri Lankarani

**Affiliations:** 1 *Research Center for Traditional Medicine and History of Medicine, Shiraz University of Medical Sciences, Shiraz, Iran*; 2 *Department of Traditional Persian Medicine, School of Medicine, Shiraz University of Medical Sciences, Shiraz, Iran*; 3 *Health Policy Research Center, Shiraz University of Medical Sciences, Shiraz, Iran*; 4 *School of Iranian Traditional Medicine, Shahid Beheshti University of Medical Sciences, Tehran, Iran*; 5 *Traditional Medicine and Materia Medica Research Center, Shahid Beheshti University of Medical Sciences, Tehran, Iran*; 6 *Department of Pharmacognosy, School of Pharmacy, Shahid Beheshti University of Medical Sciences, Tehran, Iran*; 7 *Department of Traditional Pharmacy, School of Pharmacy, Shiraz University of Medical Sciences, Shiraz, Iran*; 8 *Gastroenterohepatology Research Center, Shiraz University of Medical Sciences, Shiraz, Iran*

**Keywords:** *Functional Dyspepsia*, *Traditional Persian Medicine*, *Stomach*, *Gastrointestinal*, *Temperament*

## Abstract

**Objective:**

One of the most common global disorders is related to gastrointestinal system. Functional dyspepsia (FD) defined as upper abdominal pain and discomfort in the absence of organic ailments is a prevalent disease without any confirmed medication. The purpose of this study was to find gastric disorders which might be coincidental to FD based on traditional Persian medicine (TPM).

**Materials and Methods:**

We searched the main textbooks of TPM including Al-Havi (by Rhazes), Canon of medicine (by Avicenna), ZakhireKhawrazmshahi (by Ismail Jorjani), Moalijat-e Aghili and Makhzan Al-adviya (by Mohammad Hosein AghiliShirazi), and ExirAzam (by Hakim Azam Khan). Also, we searched Pubmed, Scopus, Science Direct, Medline, scientific information database (SID), Iranmedex and Google Scholar from 1980 to 1 August 2014 for dyspepsia, gastrointestinal disease, traditional Persian medicine, and gastric dystemperaments.

**Results:**

There is no equivalent term for FD in traditional Persian medicine although similar signs and symptoms are visible in terms like simple cold dystemperament of stomach, indigestion, and digestion debility in TPM sources. Some treatments mentioned in TPM have shown promising results in the current experimental tests.

**Conclusion:**

Finding these similarities in complementary and alternative medicine (CAM) textbooks may lead to discovering new remedies for this widespread disease.

## Introduction

Gastrointestinal diseases are very common in different populations, and many physicians spend a great deal of time and energy to treat these disorders carefully worldwide (Amini et al., 2012 ▶; Camilleri and Stanghellini, 2013 ▶; Moayyedi et al., 2011 ▶). Dyspepsia is a condition with recurrent or persistent discomfort or pain in the upper abdomen, which underlies several diseases including gastro-esophageal reflux disease (GERD), peptic ulcer disease (PUD), esophagitis, and gastric cancer (Khademi et al., 2012 ▶). Dyspepsia is a common disorder in developed countries with a worldwide prevalence of 7–34.2%; however, the prevalence of uninvestigated dyspepsia was estimated to be about 8.5% in the study of Barzkar et al. among Iranian people (Barzkar et al., 2009 ▶). Dyspepsia is very prevalent (29.9%) in Southern Iran and more prevalent in female patients (Mostaghni et al., 2009 ▶). The term “functional dyspepsia” (FD) is used when no underlying organic diseases are found in the patients with symptoms of dyspepsia through special exams and tests like upper abdominal endoscopy. Currently, there is no definite treatment for FD patients. It is a prevalent disease among different populations (Brun and Kuo, 2010 ▶; Delgado-Aros et al., 2004 ▶).

Researchers are trying hard to find new solutions for old problems arising from dysfunction in a vital system in order to decrease the burden of the disease (Farzaei et al., 2013 ▶; Pasalar et al., 2013 ▶; Schmulson and Chang, 2011 ▶). According to the principles of medicine, treatment of any disease is based on precise understanding of its pathophysiology (Balouch et al., 2014 ▶). Different medical schools put forward a special explanation for the appearance and progress of a known disease and the therapeutic options.

**Diagram 1:**
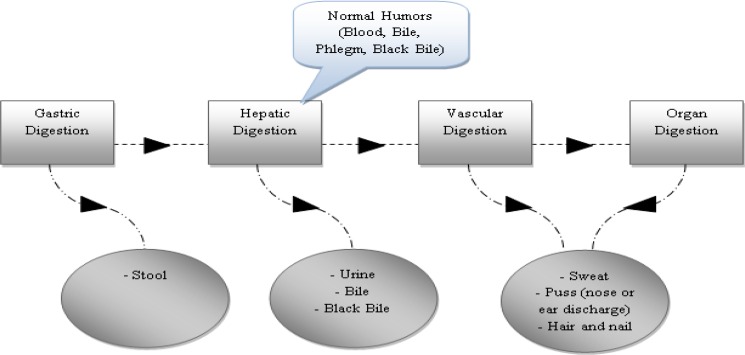
Schematic pathway of digestion and its waste materials

Traditional Persian medicine (TPM), called humoral medicine, as a famous medical school is based on four humors (bile, blood, phlegm, black bile) (Javed et al., 2009 ▶; Pasalar, 2014 ▶). Any imbalance in humoral equilibrium (dystemperament) may result in organ disorder and gastrointestinal system is not an exception (Emtiazy et al., 2013 ▶; Jorjani, 2001 ▶). TPM elites believe that gastrointestinal (GI) system has a main role in health maintenance not only for a single organ but also for the whole body. If it works properly, it will produce normal humors in a balanced quality and quantity (mezaj-e-sehhi) (Table 1) (Azam Khan, 2008 ▶). The default task for GI system is digestion, and any condition affecting four-step GI system function (Diagram 1) either in the form of food quality and quantity or its processing through alimentary canal may result in dystemperament (Avicenna, 1988 ▶; Nimrouzi and Zare, 2014 ▶). There is not any terminology describing dyspepsia in TPM resources, although the signs and symptoms have been mentioned in TPM texts repeatedly (Avicenna, 1988 ▶; Jorjani, 2001 ▶; Razi, 2000 ▶). GI diseases are the result of dystemperament (Su’emezaj), parting connections (Tafarrogh-e-ettessa'l), or both (Avicenna, 1988 ▶). Each dystemperament has its special signs and symptoms, and a comprehensive history and physical exam is necessary to detect the original organ temperament (mezaj-e-khelqati) and the acquired one (mezaj-e-âdati) (AghiliShirazi, 2008 ▶). Afterward, the therapeutic strategies set to return the organ to its default condition by health preservation principles (ossul-e hefz-o-sehheh), or/and simple medicines (daru-ye mofradeh) or/and compound medicines (daru-ye-morakkabeh) or/and physical interventions (a’mal-e-yadavi). So, in TPM school, etiology of GI disease is obvious, and this is the hakim (physician)’s duty to perceive the patients’ signs and symptoms and diagnose the disorder correctly (AghiliShirazi, 2006 ▶; Avicenna, 1988 ▶; Azam Khan, 2008 ▶). The aim of this study was to review gastric dystemperaments and its characteristics to find an equivalent terminology for dyspepsia according to TPM school.

**Table 1:** Normal temperaments of the stomach

**Normal Gastric Mezaj**	**Signs**	**Symptoms**
**Hot**	- good digestion of heavy meals (beef)- spoiling of tenuous meals (milk, chicken)	- advantage from hot-temperament foods- good appetite
**Cold**	- good digestion of light meals - problematic digestion of heavy meals	- advantage from cold-temperament foods- normal appetite
**Moist**		- low thirst- tolerance for consumption of much drinks without fullness - advantage from moist-temperament foods
**Dry**		- high thirst- advantage from a little drinks- fullness while drinking much- advantage from dry-temperament foods
**Hot and Moist**	Combination of hot and moist temperament signs and symptoms	
**Hot and Dry**	Combination of hot and dry temperament signs and symptoms	
**Cold and Moist**	Combination of cold and moist temperament signs and symptoms	
**Cold and Dry**	Combination of cold and dry temperament signs and symptoms	

## Materials and Methods

In this study, we searched some main resources of TPM scholars namely Al-Havi (by Rhazes), Canon of medicine (by Avicenna), ZakhireKhawrazmshahi (by Ismail Jorjani), Moalijat-e Aghili and Makhzan Al-adviya (by Mohammad Hosein AghiliShirazi), and ExirAzam (by Hakim Azam Khan) for gastric dystemperaments and treatments. Also, we searched Pubmed, Scopus, Science Direct, Medline, scientific information database (SID), Iranmedex, and Google Scholar from 1980 to 1 August 2014 for dyspepsia, gastrointestinal disease, traditional Persian medicine, and gastric dystemperaments. Those studies with randomized clinical trial or experimental methodology were included and reviewed by three authors. The aforementioned books were also reviewed focusing on GI system chapters by the same authors. The selected literature was discussed by all authors in several panel discussions and summarized for the manuscript draft.

## Results

According to TPM principles, the stomach is one of the most important organs because it is the main route for food entrance and digestion (Nimrouzi and Zare, 2014 ▶). The stomach is known as Ozv-e-sharifeh (respected organ) and is considered as the main cause of diseases (AghiliShirazi, 2006 ▶; Avicenna, 1988 ▶).

**Anatomy and physiology of stomach in TPM resources:**

The stomach is a muscular, hollow, dilated part of the digestion system, which is located between the esophagus (Meri) and the duodenum (Asna-ashar) (Jorjani, 2001 ▶). The stomach includes two sphincters, which keep the food contents during the completion of food digestion, the esophageal sphincter, which is not an anatomical sphincter (cardia), and the pyloric sphincter that connetcsthe stomach to the duodenum. In TPM, the cardia and pylorus are called Fam-e-me'deh and Bavvab, respectively, and the antrum is known as Qa'r-e-me'deh (Azam Khan, 2008 ▶) although the cardia is not equal to Fam-e-me’deh, unquestionably. According to TPM, the stomach has two layers, namelyneural and muscular. The muscular layer comprises three types of fibers: longitudinal fibers for digestion, diagonal for retaining, and transverse for repulsion of the ingested materials. The stomach is in the vicinity of two main organs, that is, the heart in the upper and the liver in the right side (AghiliShirazi, 2008 ▶). Avicenna believed that Hararat-e-qarizie or intrinsic heat contributes to digestion of food in the stomach. The heart is hot and dry in temperament because it has non-stop movements, and the liver is hot and moist because it contributes to digestion, development, and body homeostasis. These two organs are warmer than the stomach, transfer their intrinsic heat to the stomach and help it with meal digestion (Avicenna, 1988 ▶).

**Semiology and Pathophysiology of functional dyspepsia:**

Dyspepsia is made of two Greek words which together mean indigestion (Brun and Kuo, 2010 ▶). Based on the 2006 Rome III criteria, early satiety, postprandial fullness, upper abdominal discomfort or pain, and epigastric burning without any evidence for structural disorder are the prominent features of functional discomfort. This classification divides functional dyspepsia into two subgroups: postprandial distress syndrome (PDS) and epigastric pain syndrome (EPS) (Nwokediuko et al., 2012 ▶). FD is a heterogeneous disorder, and it seems that psychosocial factors, gastrointestinal motor abnormalities, and altered visceral sensation contribute to the pathophysiology of functional dyspepsia. About 30–70% of the patients with functional dyspepsia experience delayed gastric emptying. Impaired accommodation to the food is another frequent finding in dyspeptic patients. Visceral hypersensitivity is more prominent in patients with functional dyspepsia compared to healthy individuals and dyspeptic patients with organic causes. Some patients with functional dyspepsia develop impaired gastric and intestinal reflexes. Impaired reflex relaxation in the antral region leads to antral hypersensitivity and antral overload, and it may be a reason for occurrence of dyspeptic symptoms (Brun and Kuo, 2010 ▶; Thumshirn, 2002 ▶). The size of the food ingested and gastric emptying rate leads to aggravation of symptoms in chronic dyspeptic patients further than age and body weight. A correlation between the history of dyspepsia, headache, consumption of pickles, and stressful conditions and developing GERD was shown (Saberi-Firoozi et al., 2007 ▶). Current studies on FD pathophysiology concentrate on topics like “central processing of visceral stimuli, low-grade duodenal inflammation, and genetic predisposition” (Tack et al., 2011 ▶).

## Discussion

In TPM resources, we did not find a term equivalent to functional dyspepsia. It is unbelievable that we suppose TPM elites had no patients suffering from symptoms in favor of FD, so we should search the main textbooks of Persian medicine for symptoms like epigastric pain or postprandial fullness to solve the puzzle. Among12 types of dystemperaments arising from the stomach, there are some similarities to FD symptoms (Table 2). The most similar condition to FD is simple cold and moist dystemperament without excess humor. The signs and symptoms are relatively the same as what is mentioned in the Rome III criteria for FD. The signs that are present in physical exam include weak digestion, and symptoms like postprandial fullness (maybe equals to Seghl) and abdominal discomfort (with some symptoms somewhat equals to Herghat or Nafkh) support this theory, despite the fact that all necessary criteria are not fulfilled.

If the patient does not care about the symptoms and simple cold gastric dystemperament without excess humor goes on, a new persistent condition called tenacious dystemperament will appear (Su-e-mezajmostahkam) (AghiliShirazi, 2006 ▶; Razi, 2000 ▶). This situation produces two new entities in TPM texts: indigestion (Su-ol-hazm) and digestion debility (Za’af-ol-hazm). These disorders have signs and symptoms which are so similar to FD (Table 3) (Azam Khan, 2008 ▶; Jorjani, 2001 ▶). Based on TPM resources, prolonged dystemperament will cause tenacious dystemperament and organ debility subsequently. Continuous gastric dystemperament will result in weakness of GI system, and the digestion would be impaired. So, the resulting humors would be abnormal (Khelt-e nasaleh), and the whole body would be affected (AghiliShirazi, 2006 ▶; Razi, 2000 ▶).

In TPM, the stomach is considered as a main source of disease, and the abstinence (Parhiz) is the best remedy for all diseases. The diseases of the stomach as an individual organ and gastrointestinal tract in general, follow this rule. Tabi'at-e-modabbere (nature) or sober force of the body is considered the internal army of the body to maintain body health and defend the body in front of intrinsic and extrinsic harmful agents. Tabi'at-e-modabbere in TPM may partly be considered as an equivalent for the immune system and neuroendocrine system together (Avicenna, 1988 ▶; Nimrouzi et al., 2014 ▶).

Treatment of FD in conventional medicine is unsatisfactory, although extensive studies have been conducted recently. Life style changes and reassurance are primary treatment for those who suffer from mild symptoms of FD. For those with severe symptoms or nonresponsive to the latter treatment, proton pump inhibitors (PPIs) and prokinetics are good choices for empirical pharmacotherapy (Camilleri and Stanghellini, 2013 ▶; Feinle-Bisset and Azpiroz, 2013 ▶; Leake, 2013 ▶). Psychiatric or psychotherapist consultation and consumption of antidepressant drugs are advisable for special cases with FD diagnosis (Chou et al., 2001 ▶; Li et al., 2002 ▶; Mahadeva and Goh, 2011 ▶). Some researchers proposed surgical interventions to relieve symptoms, although some side effects may appear later. Niessen fundoplication is the gold standard treatment for anatomic correction of cardia, especially in patients with typical symptoms of heartburn and regurgitation (Oleynikov and Oelschlager, 2003 ▶). Anti-reflex surgery, however, controls the GE refluxes in afflicted patients, but some annoying symptoms such as retching and bloating appear in many patients after operation (Jolley et al, 1987 ▶).

**Table 2:** Gastric dystemperaments based on TPM resources

**Stomach Dystemperament**	**Excess Humor**	**Signs **	**Symptoms**
**Hot **		- drastic digestion- dry mouth- spoiling of tenuous food in stomach	- low appetite- smoky belching- severe thirst - dry stool- fast passage of food through stomach- advantage with cold-temperament foods- disadvantage with hot-temperament foods
**Hot and Dry **		- good digestion- dry mouth- thin body	- low appetite- severe thirst - dry stool- fast passage of food through stomach- advantage with cold-temperament foods- disadvantage with hot-temperament foods- advantage with moist-temperament foods- disadvantage with dry-temperament foods
**Hot and Dry**		- good digestion- dry mouth - stark tongue - yellowish urine- thin body- yellowish skin- spoiling of tenuous food in stomach	- low appetite- smoky belching- severe thirst - bile stool- fast passage of food through stomach- advantage with cold-temperament foods- disadvantage with hot-temperament foods- advantage with moist-temperament foods- disadvantage with dry-temperament foods- bitter mouth taste - bile-containing vomit- epigastric burning sensation after meal
**Hot and Moist**		- extra production of saliva	
**Hot and Moist**		- extra production of saliva (especially while starvation)	- normal appetite - belching with spoilt food odor- weak thirst- vomit
**Cold**		- weak digestion	- weak thirst- advantage with hot-temperament foods- disadvantage with cold-temperament foods- bloating
**Cold and Dry**		- severe dry tongue- thin body	- postprandial fullness- food odor belching- advantage from watery foods- bloating
**Cold and Dry**		- weak digestion- splenomegaly	- postprandial fullness- gorge (waste material)- black-bile containing vomit - epigastric burning sensation before meal- severe bloating
**Cold and Moist**		- extra production of saliva- lazy movements- phlegmatic face	- postprandial fullness- food odor belching- weak thirst - disadvantage from moist-temperament foods - fast passage of food through stomach- loose stool- bloating
**Cold and Moist**		- weak digestion	- weak appetite- pseudo-thirst- advantage from hot-temperament foods- advantage from dry-temperament foods- vomit
**Dry **		- severe dry tongue- thin body	- severe thirst- advantage from watery foods
**Moist **		- extra production of saliva	- weak thirst- advantage from dry-temperament foods- disadvantage from moist-temperament foods- fast passage of food through stomach

**Table 3:** Gastric disorders arising from stomach dystemperament

Gastric ailment	Signs	Symptoms
**Indigestion **	- incomplete digestion- subcostal bulging	- malodor stool- malodor (sour or bitter) smoky belching- heartburn
**Digestion debility**	- delayed food passage from stomach	- postprandial fullness- malodor belching

Complementary and alternative medicine (CAM) modalities have multiple therapeutic options for relief of FD symptoms, and herbal medicines and natural products are of great importance (Kav, 2009 ▶; Thompson Coon and Ernst, 2002 ▶). Celery (*Apiumgraveolens*), radish (*Raphinussativus* L.), rocket (*Eruka sativa*), and marjoram (*Origanummajorana* L.) demonstrated anti-ulcer effect in experimental investigations (Al-Howiriny et al., 2009 ▶, 2010; Alqasoumi et al., 2009 ▶; Devaraj et al., 2011 ▶), and the aqueous methanolic extract of pomegranate (*Punicagranatum*) showed such an activity in wistar pylorus ligated rats (Alam et al., 2010 ▶). Anti-inflammatory, anti-*H.pylori*, anti-peptic ulcer, antioxidant, cytoprotective, and wound healing effects of fruits like amla (*Phyllanthusemblica*), grape (*Vitisvinifera*), and nutmeg (*Myristicafragrans*), which have been advised in TPM resources, have been proven by modern investigations(Farzaei et al., 2013 ▶). There are numerous well-designed studies investigating the effect of single medicinal plants like red pepper (*Capsicum annuum*), liquorice (*GlycyrrhizaGlabra*), and black caraway (*Niggella sativa*) or compound medicinal plants like Jollab on the elimination of symptoms of FD in patients with promising results (Bone, and Mills, 2013; Bortolotti et al., 2002 ▶; Pasalar et al., 2015 ▶; Raveendra et al., 2012 ▶; Rosch et al, 2006 ▶). Some of these remedies have been suggested for the treatment of cold gastric dystemperament without excess humor in TPM texts and pharmacopeia (AghiliShirazi, 2009 ▶; Azam Khan, 2008 ▶).

The treatment of gastric temperaments either new or tenacious form is different from what is explained above in conventional medicine, although recommendations for changing dietary habits and life style in addition to promotion of the mental health are lookalike, and some are advised in new studies (Avicenna, 1988 ▶; Feinle-Bisset and Azpiroz, 2013 ▶). For instance, right decubitus position in the first postprandial hour and then changing to the left decubitus position decrease the chance of GE reflux in infants with GERD (van Wijk et al., 2007 ▶). This advice is found in TPM books frequently (AghiliShirazi, 2008 ▶; Razi, 2000 ▶). Regulation of dietary habits (time, amount, order, quality, and temperament of ingesting food or drink) is of great importance in this regard as it may be curative by itself (Avicenna, 1988 ▶; Razi, 2000 ▶). The next step in TPM therapeutic protocol for gastric dystemperament is to use a proper herbal drug in different dosage forms. If excess humor is present with gastric temperament, the practitioner should purify (Tanghiye) the stomach through a safe route. When prolonged or tenacious dystemperament takes place and organ debility occurs subsequently, reinforcement (Taghviyat) of the stomach is the last stage of the treatment process (AghiliShirazi, 2008 ▶; Azam Khan, 2008 ▶; Shirzad et al., 2013 ▶). There is a long list of tonic agents for stomach in TPM pharmacopeia, and some of them have successfully passed experimental tests. Cinnamon (*Cinnamomumzeylanicum*) showed promising effects on the stomach in rats (AghiliShirazi, 2009 ▶; Rafatullah et al., 2011 ▶).

GI problems are among the most common diseases worldwide. Functional dyspepsia is a heterogeneous disorder with empirical treatments, and CAM practitioners are trying to put forward optimal remedies for it through well-designed researches. TPM school has its unique pathophysiology and therapies for GI diseases. Finding equivalent terminology for FD and reviewing the suggested medications may be so beneficial to fight this prevalent disorder. Promising results in this field trigger “the glimmers of hope” for future.

## Conflict of interest

The authors certify that there is no actual or potential conflict of interest in relation to this article. 
